# Differences in the roles of types 1 and 2 diabetes in the susceptibility to the risk of fracture: a systematic review and meta-analysis

**DOI:** 10.1186/s13098-021-00687-8

**Published:** 2021-08-16

**Authors:** Jiaqing Dou, Jing Wang, Qiu Zhang

**Affiliations:** 1 Department of Endocrinology, The First Affiliated Hospital of Anhui Medical University, 218 Jixi Road, Shushan District, Hefei, 230022 Anhui China; 2 Department of Endocrinology, Chaohu Affiliated Hospital of Anhui Medical University, Hefei, China

**Keywords:** Diabetes Mellitus, Type 1, Diabetes Mellitus, Type 2, Fractures, Bone, Meta-analysis

## Abstract

**Background:**

Diabetes mellitus (DM) causes excess risk of fracture at varied sites. Whereas, the difference between the roles of types 1 DM (T1DM) and 2 DM (T2DM) diabetes in the risk of fractures remains limited and inconclusive. We, therefore, conducted a meta-analysis to assess the differences for the associations of T1DM and T2DM with the risk of fractures.

**Methods:**

We systematically searched PubMed, Embase, and the Cochrane library for eligible studies until May 2021. The odds ratios (ORs) with 95% confidence intervals (CIs) were used to calculate the pooled effect estimates for the associations of T1DM and T2DM with the risk of fractures using the random-effects model. An indirect comparison results for the ratio of OR (ROR) with 95% CI were also applied to assess the difference between T1DM and T2DM with the risk of fractures.

**Results:**

Twenty-two cohort studies involving a total of 6,484,851 individuals were selected for meta-analysis. We noted that T1DM was associated with an increased risk of all fractures (OR: 1.72; 95% CI 1.36–2.19; *P* < 0.001), and fractures at the hip (OR: 4.01; 95% CI 2.90–5.54; *P* < 0.001), upper arm (OR: 2.20; 95% CI 1.61–3.00; *P* < 0.001), ankle (OR: 1.97; 95% CI 1.24–3.14; *P* = 0.004), and vertebrae (OR: 2.18; 95% CI 1.85–2.57; *P* < 0.001). Moreover, T2DM induced excess risk to all fractures (OR: 1.19; 95% CI 1.09–1.31; *P* < 0.001), including fractures at the hip (OR: 1.25; 95% CI 1.15–1.35; *P* < 0.001), upper arm (OR: 1.42; 95% CI 1.20–1.67; *P* < 0.001), and ankle (OR: 1.15; 95% CI 1.01–1.31; *P* = 0.029). Furthermore, we noted that T1DM versus T2DM was associated with greater risk to all fractures (ROR: 1.45; 95% CI 1.12–1.87; *P* = 0.005), including fractures at the hip (ROR: 3.21; 95% CI 2.30–4.48; *P* < 0.001), upper arm (ROR: 1.55; 95% CI 1.09–2.20; *P* = 0.015), and ankle (ROR: 1.71; 95% CI 1.06–2.78; *P* = 0.029).

**Conclusions:**

This study found that T1DM caused an excess risk to all fractures, including fractures at the hip, upper arm, and ankle than T2DM. Further studies should therefore be conducted to directly compare the differences between T1DM and T2DM with the risk of fractures at various sites.

**Supplementary Information:**

The online version contains supplementary material available at 10.1186/s13098-021-00687-8.

## Background

The prevalence of diabetes has increased from 4.7–8.5% worldwide, directly resulting in approximately 1.6 million deaths in 2016 [1]. The inadequate control of diabetes affects the patient’s quality of life through diabetes-specific symptoms and microvascular complications [2, 3]. Diabetes and its complications are also considered a global burden, therefore, reducing the disease burden of diabetes is an important goal of medical care societies and health policymakers [4, 5]. Patients with diabetes are susceptible to excess risk of cardiovascular disease, neuropathy, nephropathy, retinopathy, and mortality [6]. Moreover, the rapidly increasing diabetes prevalence was also parallel with an increase in osteoporotic fractures [7].

Numerous studies have found a positive association of diabetes with the risk of fractures [8–14], and the potential reason for the association between diabetes and fractures included increased frequency of falling, cortical porosity, microvascular disease, and high levels of advanced glycation end-products [15–17]. The National Osteoporosis Foundation guidelines, therefore, suggested that screening for osteoporosis should be conducted for general women aged ≥ 65 years and men aged ≥ 70 years to prevent the morbidity and mortality related to fractures [18]. However, the strength of the association of type 1 diabetes mellitus (T1DM) and type 2 DM (T2DM) with the risk of fractures remains controversial. We, therefore, conducted a systematic review and meta-analysis of cohort studies to assess the differences between the associations of T1DM and T2DM with the risk of fracture at various sites. Moreover, whether study design and gender affected this difference was also evaluated.

## Methods

### Data sources, search strategy, and selection criteria

The Meta-analysis Of Observational Studies in Epidemiology protocol was used to conduct and report this systematic review and meta-analysis [19]. On the basis of this protocol, cohort studies that investigated the role of T1DM or T2DM with the risk of fractures were eligible in our study. The databases of PubMed, Embase, and the Cochrane library were searched for eligible studies from their inception up to May 2021. The following terms were used as medical subject headings or text words: (“diabetes” OR “diabetes mellitus” OR “glucose” OR “glycated hemoglobin”) AND (“fractures, spontaneous” OR “osteoporotic fractures” OR “fractures, compression” OR “fracture”). The reference lists of potentially relevant articles were also manually reviewed for additional new eligible studies.

The literature search and study selection were conducted independently by 2 reviewers, and face-to-face discussions were used to settle disagreements until a consensus was reached. A study was included if they fulfilled the following inclusion criteria: (1) Study design: prospective or retrospective cohort studies; (2) Participants: general population; (3) Exposure and control: T1DM, T2DM, and non-DM population; (4) Outcome: all fracture, or fractures at hip, distal forearm, upper arm, ankle, and vertebrae; and (5) all the studies should have reported the effect estimates for the role of T1DM or T2DM with the risk of fractures. This study did not contain any human or life participants, therefore, ethics approval and informed consent were not applicable.

### Data collection and quality assessment

Information from included studies contained the first author or study group’s name, publication year, region, study design, sample size and number of DM, mean age, male proportion, smoking proportion, body mass index (BMI), DM type, follow-up duration, adjusted factors, and reported effect estimates. The quality of the individual studies were also assessed using the Newcastle–Ottawa Scale (NOS), which is widely used for assessing the quality of observational studies in a meta-analysis. The scoring system for each study ranged from 0–9 [20]. Studies having between 7 and 9 stars were regarded as high-quality. Data extraction and quality assessment were also independently performed by 2 reviewers, and any inconsistency was resolved and adjudicated by an additional reviewer during reading of the full-text of studies.

### Statistical analysis

The role of T1DM and T2DM in the risk of fractures was calculated based on the effect estimates (relative risk, hazard ratio, or odds ratio [OR]) using the 95% confidence intervals (CIs) in individual studies. The pooled OR was also calculated using the random-effects models, which considered the underlying variations across included studies [21, 22]. Then, the OR ratio (ROR) with a 95% CI was estimated on the basis of specific ORs, and 95% CIs were taken for T1DM and T2DM studies having risk of fractures [23]. Heterogeneity for each investigated outcome was also assessed using the *I*^*2*^ and Q statistic, and significant heterogeneity was defined as *I*^*2*^ > 50.0% or *P* < 0.10 [24, 25]. Subgroup analyses for the differences of T1DM and T2DM with the risk of fractures were also assessed on the basis of the study design, and gender. Similarly, publication bias was evaluated as well using qualitative and quantitative methods, including funnel plots, Egger, and Begg tests [26, 27]. The 2-sided inspection level for pooled results was adopted, and statistical significance was set at *P* < 0.05. Also, all the analyses in our study were performed using the STATA (version 10.0; STATA Corporation, College Station, TX, USA) software.

## Results

### Literature search

**Fig. 1:**
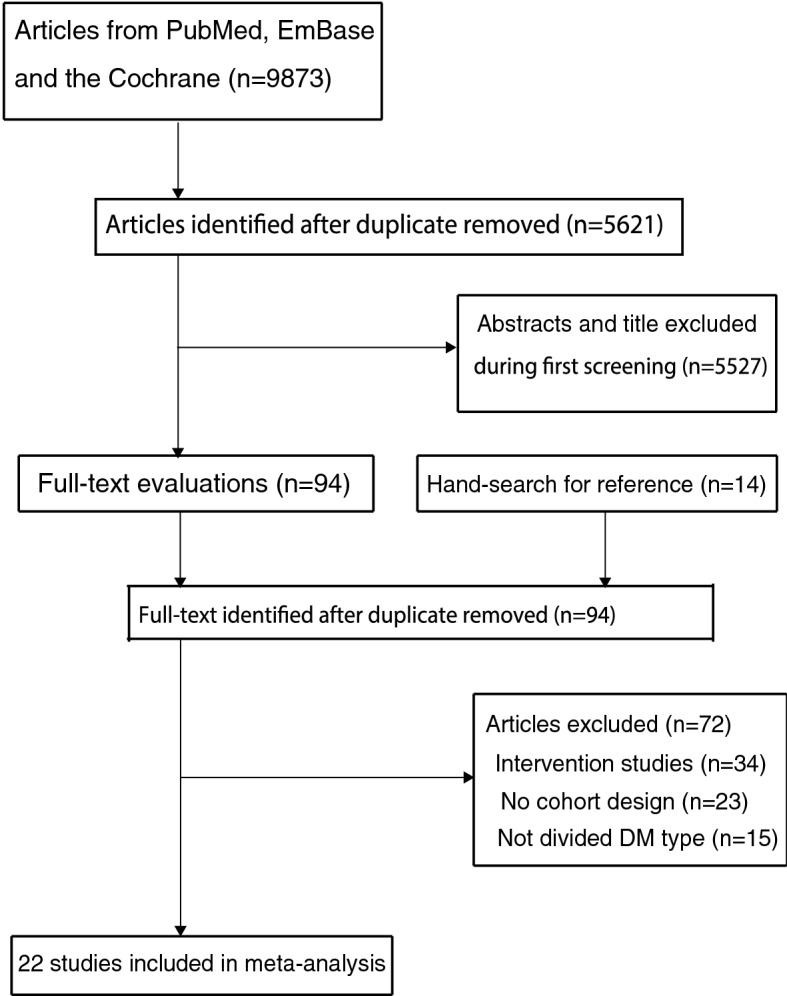
Details regarding literature search and study selection

A total of 9873 articles were identified from initial electronic searches, and 5621 articles were retained after duplicate articles were removed. A total of 5527 articles were further removed because these studies reported irrelevant topics. The remaining 94 studies were then retrieved for further full-text evaluations. Of these 94 remaining studies, 72 of them were excluded because they were intervention studies (n = 34); not cohort designs (n = 23); and did not differentiate DM types (n = 15). Reviewing the references of relevant articles found additional 14 potentially included studies, including all studies contained in electronic searches. Finally, 22 cohort studies were selected for the final meta-analysis [28–49] (Fig. 1).

### Study characteristics

Of the 22 included studies, 14 studies were prospective cohort studies, whereas the remaining 8 studies were retrospective cohort studies. The characteristics of identified studies and individuals are shown in Table 1. A total of 6,484,851 individuals and 766,610 patients with DM were identified from the 22 studies. Nine studies reported the role of T1DM with the risk of fractures, and 20 studies reported the role of T2DM with the risk of fractures. The follow-up duration ranged from 1.3 to 20.0 years. Also, 9 studies contained only females. Similarly, study quality assessment using the NOS-scoring method showed that 3 studies had 9 stars, 7 studies had 8 stars, 8 studies had 7 stars, and the remaining 4 studies had 6 stars.

**Table 1:** The characteristics of identified studies and involved participants

Study	Region	Study design	Sample size/DM	Mean age (years)	Male (%)	Smoker (%)	BMI (kg/m^2^)	DM type	Follow-up (years)	Adjusted factors	NOS score
NTHS 1999 [28]	Norway	Prospective	35,444/1850	50.0–74.0	47.5	30.4	NA	I and II	9.0	Age, BMI and daily smoking	8
IWHS 2001 [29]	USA	Prospective	32,089/1729	61.6	0.0	15.0	26.9	I and II	9.6	Age, smoking, estrogen use, BMI, and WTHR	9
BMES 2001 [30]	Australia	Prospective	3654/216	66.2	43.3	NA	NA	II	5.0	Age, sex, and BMI	7
SOF 2001 [31]	USA	Prospective	9754/657	71.0	0.0	NA	26.2	II	9.4	Age, BMI, calcaneal BMD, height, height loss since age 25, contrast sensitivity, walking speed, consumed alcohol in past year, resting pulse, mother fractured hip, on feet < 4 h a day, use of long-acting benzodiazepines, and calcium intake	9
H-EPESE 2002 [32]	USA	Prospective	2884/690	71.8	42.1	42.1	NA	II	7.0	Age, gender, BMI, ever smoked, previous stroke, lower extremity functional ability, and distance vision	7
SIR 2005 [33]	Sweden	Retrospective	24,605/24,605	20.7	51.0	NA	NA	I	9.9	Age, sex, and calendar-period-matched general population from the entire Swedish inpatient registry	6
Dobnig 2006 [34]	Australia	Prospective	1664/583	> 70.0	0.0	NA	NA	II	2.0	Age and weight	6
NHS 2006 [35]	USA	Prospective	109,983/8640	56.3	0.0	17.9	26.0	I and II	20.0	Age, BMI, physical activity, menopausal status and estrogen use, smoking and daily intake of calcium, vitamin D, and protein	9
Tromsø 2006 [36]	Norway	Prospective	27,159/455	47.0	47.7	37.0	25.5	I and II	6.0	Age, BMI, smoking, and metabolic features	8
WHI 2006 [37]	USA	Prospective	93,676/5285	63.4	0.0	6.2	NA	II	7.0	Age, ethnicity, weight, height; time-dependent history of falls, previous fracture, history of osteoporosis, trouble seeing at baseline, alcohol or tobacco use, calcium and vitamin D intake, exercise, bisphosphonate, estrogen, steroid, insulin, SERM, or thyroid hormone use	8
Melton 2008 [38]	USA	Retrospective	1964/1964	61.7	51.0	NA	NA	II	11.8	Age, BMI, calcaneal BMD, or a host of other osteoporosis risk factors	6
CHS 2011 [39]	USA	Prospective	5641/1456	72.8	42.0	12.0	26.7	II	10.9	Age, sex, race, BMI, AAI < 0.9	8
Jung 2012 [40]	Korea	Retrospective	2282/1268	61.0	0.0	NA	25.0	II	7.0	Age	7
Rotterdam 2013 [41]	Netherland	Prospective	4135/420	68.4	40.6	25.0	26.4	II	12.2	Age, sex, height, weight, and femoral neck BMD	8
SCI-DC 2014 [42]	UK	Retrospective	3,801,874/201,874	20.0–84.0	NA	NA	NA	I and II	NA	Age, calendar year, SIMD, and for the overall estimate, an SIMD-age interaction	7
SIDIAP 2015 [43]	Spain	Prospective	171,931/58,483	62.6	56.5	15.6	29.3	II	2.6	BMI, previous fracture, oral corticoids	7
THIN 2015 [44]	UK	Retrospective	334,266/30,394	34.0	56.1	26.7	25.5	I	5.7	Exposure to steroid medication, history of prior fracture, and presence of chronic kidney disease	6
Manitoba 2016 [45]	Canada	Retrospective	57,938/8840	64.3	0.0	NA	27.1	II	7.2	FRAX scores, burden of comorbidity, falls, prescription osteoporosis treatments, and insulin therapy	8
FRAILCO 2017 [46]	Sweden	Prospective	428,305/84,702	80.8	42.4	NA	25.4	I and II	1.3	Age, sex, weight, height, previous fracture, RA, glucocorticoid, alendronate use, and CCI, and self-reported known fall injury	8
Holm 2018 [47]	Denmark	Retrospective	6285/229	61.1	0.0	28.0	23.4	II	5.8	Baseline age group, BMI group, modified Charlson index, estrogen deficiency, prevalent hyperthyroidism, RA, CPD, MOF, former osteoporosis treatment, glucocorticoid use, calcium intake, family fracture history, current smoking, exercise alcohol related diagnoses and current use of ACE, ANGII, loop, thiazide, SSRI, TCA	7
DNPR 2019 [48]	Denmark	Retrospective	1,328,336/332,084	59.0	52.9	NA	NA	I and II	6.0	Age, sex, previous fracture, anti-osteoporosis medication	7
PK-VF 2019 [49]	China	Prospective	982/186	62.0	0.0	NA	26.0	II	5.2	Age, YSM, BMI, LS BMD, and any previous fractures	7

### All fracture

**Fig. 2:**
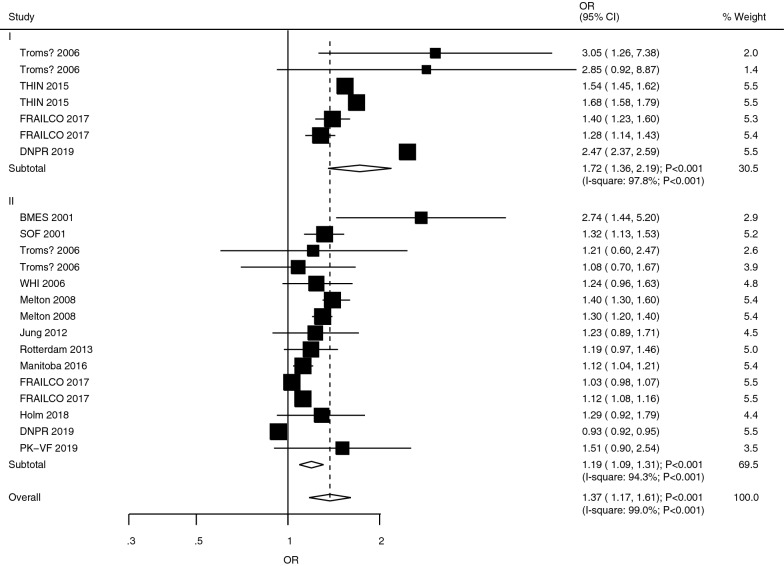
The role of T1DM and T2DM in the risk of all fractures

The studies assessing the role of T1DM and T2DM in the risk of all fractures were reported in studies 4 and 12 studies, respectively (Fig. 2). We noted that both T1DM (OR: 1.72; 95%CI 1.36–2.19; *P* < 0.001) and T2DM (OR: 1.19; 95% CI 1.09–1.31; *P* < 0.001) were associated with an increased risk of all fractures. Moreover, there was a significant heterogeneity in the role of T1DM (*I*^*2*^ = 97.8%; *P* < 0.001) and T2DM (*I*^*2*^ = 94.3%; *P* < 0.001). We also noted from the selected studies that the risk of all fractures in T1DM patients was significantly higher than in T2DM patients (ROR: 1.45; 95% CI 1.12–1.87; *P* = 0.005; Table 2). Similarly, subgroup analyses found that excess risk of fractures in T1DM patients existed during pooled prospective cohort studies (ROR: 1.21; 95%CI 1.00–1.46; *P* = 0.050), retrospective cohort studies (ROR: 1.56; 95%CI 1.09–2.24; *P* = 0.015), or studies that reported both male and female (ROR: 1.99; 95%CI 1.40–2.83; *P* < 0.001) (Table 3). Also, no significant publication bias to all fractures was observed (*P*-value for Egger: 0.075; *P*-value for Begg: 0.535; Additional file 1).

**Table 2:** The difference for the role of T1DM and T2DM with the risk of fracture

Fracture sites	DM type	OR and 95% CI	*P* value	*I*^*2*^ (%)/*P*_*Q statistic*_	Ratio for OR	*P* value between T1DM and T2DM
All	T1DM	1.72 (1.36–2.19)	< 0.001	97.8/< 0.001	1.45 (1.12–1.87)	0.005
	T2DM	1.19 (1.09–1.31)	< 0.001	94.3/< 0.001		
Hip	T1DM	4.01 (2.90–5.54)	< 0.001	95.2/< 0.001	3.21 (2.30–4.48)	< 0.001
	T2DM	1.25 (1.15–1.35)	< 0.001	86.7/< 0.001		
Distal forearm	T1DM	1.39 (0.70–2.77)	0.344	86.7/0.001	1.48 (0.73–2.98)	0.274
	T2DM	0.94 (0.82–1.07)	0.348	58.8/0.007		
Upper arm	T1DM	2.20 (1.61–3.00)	< 0.001	72.6/0.026	1.55 (1.09–2.20)	0.015
	T2DM	1.42 (1.20–1.67)	< 0.001	81.2/< 0.001		
Ankle	T1DM	1.97 (1.24–3.14)	0.004	29.3/0.234	1.71 (1.06–2.78)	0.029
	T2DM	1.15 (1.01–1.31)	0.029	0.0/0.886		
Vertebrae	T1DM	2.18 (1.85–2.57)	< 0.001	–	1.50 (0.83–2.72)	0.177
	T2DM	1.45 (0.82–2.56)	0.200	98.5/< 0.001

**Table 3:** Subgroup analyses for all fracture and hip fracture according to study design and gender

Outcomes	Factors	Groups	DM type	OR and 95% CI	*P* value	*I*^*2*^ (%)/*P*_*Q statistic*_	Ratio for OR	*P* value between T1DM and T2DM
All fracture	Study design	Prospective	T1DM	1.40 (1.18–1.65)	< 0.001	50.6/0.108	1.21 (1.00–1.46)	0.050
			T2DM	1.16 (1.07–1.27)	< 0.001	68.9/0.001		
		Retrospective	T1DM	1.86 (1.36–2.53)	< 0.001	99.0/< 0.001	1.56 (1.09–2.24)	0.015
			T2DM	1.19 (0.99–1.43)	0.060	96.6/< 0.001		
	Gender	Male	T1DM	1.50 (1.33–1.70)	< 0.001	51.2/0.129	1.25 (0.92–1.69)	0.147
			T2DM	1.20 (0.91–1.58)	0.200	93.0/< 0.001		
		Female	T1DM	1.52 (1.17–1.97)	0.002	88.9/< 0.001	1.27 (0.97–1.66)	0.085
			T2DM	1.20 (1.12–1.28)	< 0.001	54.2/0.026		
		Both	T1DM	2.47 (2.36–2.58)	< 0.001	–	1.99 (1.40–2.83)	< 0.001
			T2DM	1.24 (0.87–1.75)	0.232	87.8/< 0.001		
Hip fracture	Study design	Prospective	T1DM	4.56 (2.49–8.34)	< 0.001	91.5/< 0.001	3.28 (1.76–6.10)	< 0.001
			T2DM	1.39 (1.21–1.60)	< 0.001	80.6/< 0.001		
		Retrospective	T1DM	3.88 (2.68–5.61)	< 0.001	96.0/< 0.001	3.43 (2.35–5.03)	< 0.001
			T2DM	1.13 (1.03–1.24)	0.010	89.3/< 0.001		
	Gender	Male	T1DM	3.95 (2.10–7.43)	< 0.001	95.7/< 0.001	3.95 (2.09–7.46)	< 0.001
			T2DM	1.00 (0.93–1.08)	0.946	36.5/0.178		
		Female	T1DM	4.76 (2.66–8.52)	< 0.001	95.9/< 0.001	3.33 (1.83–6.05)	< 0.001
			T2DM	1.43 (1.25–1.64)	< 0.001	88.2/< 0.001		
		Both	T1DM	2.41 (2.20–2.65)	< 0.001	–	2.15 (1.80–2.58)	< 0.001
			T2DM	1.12 (0.96–1.31)	0.136	41.0/0.132		

### Hip fracture

**Fig. 3:**
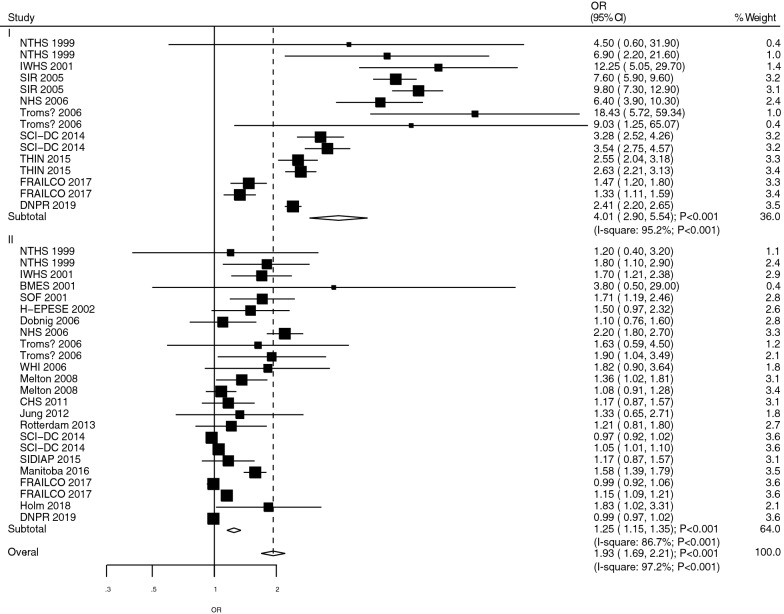
The role of T1DM and T2DM in the risk of hip fracture

The studies that reported the role of T1DM and T2DM in hip fracture risk were studies 9 and 19, respectively (Fig. 3). The summarized results indicated that T1DM (OR: 4.01; 95%CI 2.90–5.54; *P* < 0.001) and T2DM (OR: 1.25; 95%CI 1.15–1.35; *P* < 0.001) were associated with an increased risk of hip fracture. Also, there was a significant heterogeneity in the reports for the role of T1DM (*I*^*2*^ = 95.2%; *P* < 0.001) and T2DM (*I*^*2*^ = 86.7%; *P* < 0.001) in these fractures. Patients with T1DM were associated with excessive risk of hip fracture than those with T2DM (ROR: 3.21; 95%CI 2.30–4.48; *P* < 0.001; Table 2). Also, subgroup analyses found that a significant difference between T1DM and T2DM was associated with the risk of hip fracture in all subgroups (Table 3). In contrast, the Begg test did not find any significant publication bias for hip fracture (*P* = 0.856), while the Egger-test found significant publication bias (*P* < 0.001) (Additional file 1).

### Distal forearm fracture

**Fig. 4:**
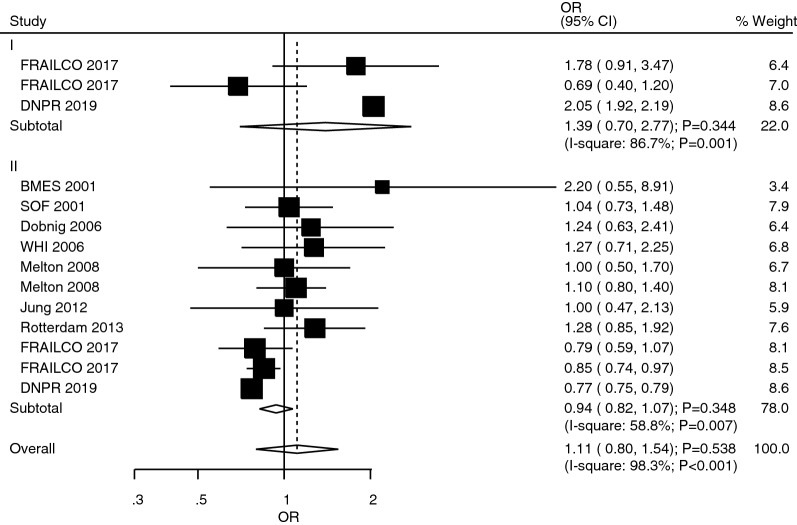
The role of T1DM and T2DM in the risk of distal forearm fracture

The studies that reported the role of T1DM and T2DM in distal forearm fracture risk were studies 2 and 9, respectively (Fig. 4). We noted that T1DM (OR: 1.39; 95%CI 0.70–2.77; *P* = 0.344) and T2DM (OR: 0.94; 95%CI 0.82–1.07; *P* = 0.348) were not associated with the risk of distal forearm fracture. However, there was significant heterogeneity in the role of T1DM (*I*^*2*^ = 86.7%; *P* = 0.001) and T2DM (*I*^*2*^ = 58.8%; *P* = 0.007) in this fracture. Also, the risk of distal forearm fracture in relation to T1DM and T2DM was not statistically significant (ROR: 1.48; 95%CI 0.73–2.98; *P* = 0.274; Table 2). Similarly, no significant publication bias to distal forearm fractures was observed (*P*-value for Egger: 0.358; *P*-value for Begg: 0.584; Additional file 1).

### Upper arm fracture

**Fig. 5:**
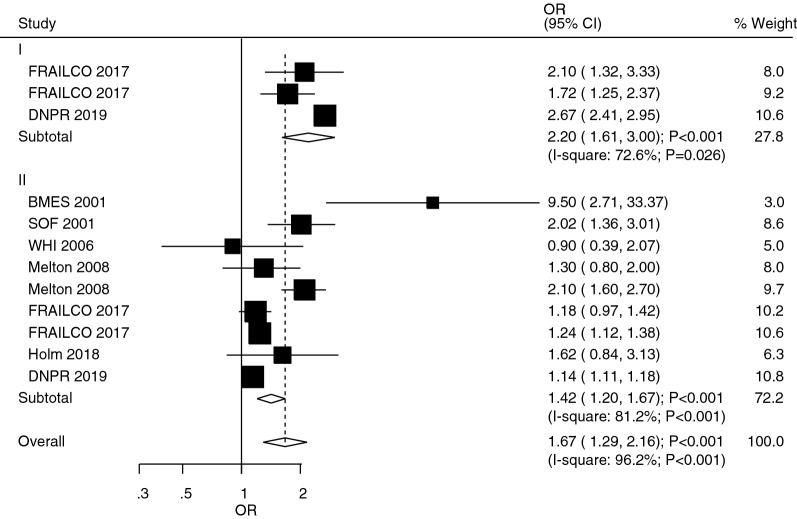
The role of T1DM and T2DM in the risk of upper arm fracture

The studies that reported the role of T1DM and T2DM in upper arm fracture risk were studies 2 and 7, respectively (Fig. 5). A summary of the results indicated that T1DM (OR: 2.20; 95%CI 1.61–3.00; *P* < 0.001) and T2DM (OR: 1.42; 95%CI 1.20–1.67; *P* < 0.001) were associated with an increased risk of upper arm fracture. Significant heterogeneity was also observed for the role of T1DM (*I*^*2*^ = 72.6%; *P* = 0.026) and T2DM (*I*^*2*^ = 81.2%; *P* < 0.001). From the results, T1DM patients showed an excessive risk of upper arm fracture than T2DM patients (ROR: 1.55; 95%CI 1.09–2.20; *P* = 0.015; Table 2). However, there was no significant publication bias to upper arm fracture (*P*-value for Egger: 0.117; *P*-value for Begg: 0.837; Additional file 1).

### Ankle fracture

**Fig. 6:**
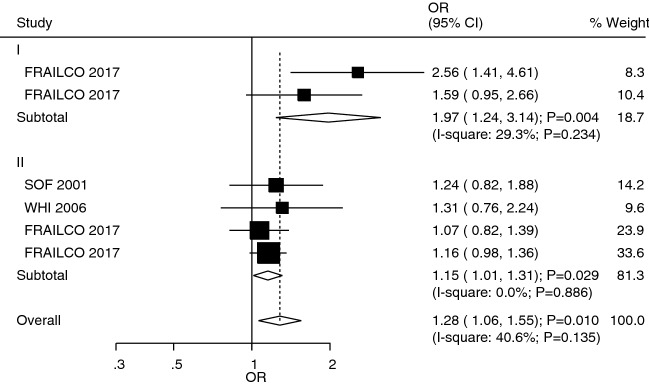
The role of T1DM and T2DM in the risk of ankle fracture

The studies that reported the role of T1DM and T2DM in ankle fracture risk were reported in studies 1 and 3, respectively (Fig. 6). We noted that T1DM (OR: 1.97; 95%CI 1.24–3.14; *P* = 0.004) and T2DM (OR: 1.15; 95%CI 1.01–1.31; *P* = 0.029) was associated with an increased risk of ankle fracture. However, no significant heterogeneity was observed across included studies for the role of T1DM (*I*^*2*^ = 29.3%; *P* = 0.234) and T2DM (*I*^*2*^ = 0.0%; *P* = 0.886). Patients with T1DM were also associated with an increased risk of ankle fracture than those with T2DM (ROR: 1.71; 95%CI 1.06–2.78; *P* = 0.029; Table 2). Similarly, no significant publication bias to ankle fracture was observed (*P*-value for Egger: 0.109; *P*-value for Begg: 0.060; Additional file 1).

### Vertebrae fracture

**Fig. 7:**
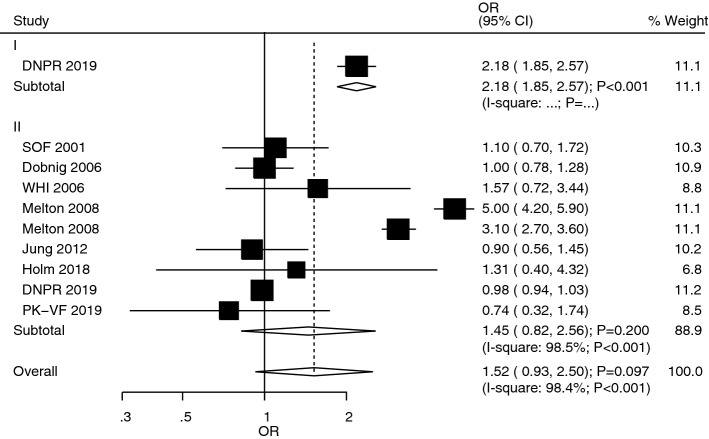
The role of T1DM and T2DM in the risk of vertebrae fracture

The studies that reported the role of T1DM and T2DM in vertebrae fracture risk were studies 1 and 8, respectively (Fig. 7). We noted that T1DM was associated with an increased risk of vertebrae fracture (OR: 2.18; 95%CI 1.85–2.57; *P* < 0.001), whereas no significant association of T2DM in vertebrae fracture risk (OR: 1.45; 95%CI 0.82–2.56; *P* = 0.200) was observed. Also, no significant heterogeneity for the role of T2DM (*I*^*2*^ = 98.5%; *P* < 0.001) was reported. Furthermore, the association between the risk of vertebrae fracture in T1DM and T2DM patients, respectively, was notstatistically significant (ROR: 1.50; 95%CI 0.83–2.72; *P* = 0.177; Table 2). Also, no significant publication bias existed in relation to vertebrae fracture (*P*-value for Egger: 0.267; *P*-value for Begg: 1.000; Additional file 1).

## Discussion

This study compared the strengths of T1DM and T2DM roles in relation to the risk of fractures. A total of 6,484,851 individuals and 766,610 patients with DM from 22 cohort studies were identified in this comprehensive quantitative meta-analysis. This study found that T1DM was associated with an increased risk of all fractures, including fractures at the hip, upper arm, ankle, and vertebrae, whereas T2DM caused excess risk of all fractures, including fractures at the hip, upper arm, and ankle. Moreover, patients with T1DM were associated with an increased risk of all fractures, hip, upper arm, and ankle fractures than those with T2DM. Furthermore, significant differences in the risk of all fractures between patients having T1DM and T2DM were mainly observed in the subgroups of prospective cohort studies, retrospective cohort studies, or studies that reported both males and females. Finally, the risk of hip fracture in T1DM patients was significantly higher than in T2DM in all predefined subgroups.

A prior meta-analysis conducted by Vestergaard et al. found that T1DM and T2DM patients were associated with an increased risk of hip fracture. An increase in the relative risk of patients with T1DM was also observed to be significantly higher than those with T2DM. Moreover, bone mineral density was increased in patients with T2DM but decreased in patients with T1DM [50]. Janghorbani et al. conducted a meta-analysis based on 2 case-controls and 14 cohort studies and found similar conclusions. Previous studies have indicated that T2DM was weakly associated with fractures at other sites [51]. However, these studies focused on the risk of hip fracture between T1DM and T2DM patients, but did not compare the strength of T1DM and T2DM with the risk of fracture at various sites. Therefore, this study was conducted to update the knowledge about discrepancies in fracture risk between T1DM and T2DM patients.

From this study, it was also found both T1DM and T2DM patients was associated with an increased risk of fractures at most sites. Moreover, the risk of all fractures, including fractures at the hip, upper arm, and ankle in T1DM patients was significantly higher than in T2DM patients. This observation proposes a potential role of T1DM in skeletal fragility, including deficits in bone mineral density, bone geometry, bone microarchitecture, and biomechanical properties [52–54]. Furthermore, the role of T2DM in the risk of fracture is proposed to be because of lower levels of bone turnover markers with reduced bone formation [55, 56]. Additionally, patients with T1DM were associated with an increased risk of all fractures, hip, upper arm, and ankle fractures than those with T2DM. The potential reason for this observation is proposed to be that T2DM patients presented higher body weight and BMI than those with T1DM patients, while the fracture in T2DM patients was due to sustained higher traumatic load and soft-tissue energy absorption in obese patients [50]. Finally, the changes in body mineral density in T1DM and T2DM patients differed, which caused the observed varying fracture risk [51].

Subgroup analyses found significant differences that existed between T1DM and T2DM patients, resulting in the risk of all fractures, including hip fracture in most of the subgroups. The risk of all fractures between T1DM and T2DM patients were not also observed in the studies that focused on males and females. This observation is because of (1) the all-fracture risk between T1DM and T2DM that was balanced by fracture at other sites. Therefore, T1DM and T2DM did not affect the risk of distal forearm; (2) the imbalance characteristics between T1DM and T2DM patients, which affected the risk of fractures; and (3) the adjusted factors between T1DM and T2DM that differed, thereby affecting the risk of fracture.

The limitations of this study should be acknowledged. First, this study contained both prospective and retrospective cohort studies, and the selection or recall biases is proposed to be biases on the risk of fracture. Second, the difference between T1DM and T2DM associated with the risk of fractures at varied sites was based on indirect comparison evidence, and various adjusted factors resulted in the risk of fractures. Third, the role of T1DM in the risk of fracture was reported in a smaller number of included studies, and the power to detect potential differences affected the comparison results. Fourth, subgroup analyses were conducted based on study design and gender, but the differences based on other characteristics were not conducted. Fifth, the severity of DM was not addressed, which is proposed to play an important role in subsequent fracture risk. Finally, inherent limitations for meta-analysis of published articles, including inevitable publication bias and the restricted detailed analyses also posed a limitation to this study.

## Conclusions

This study found that T1DM and T2DM induced excess risk of fractures at most sites. Moreover, T1DM patients were associated with an increased risk of all fractures, including fractures at the hip, upper arm, and ankle than T2DM patients. Further, large-scale prospective studies should thus be conducted to directly compare the differences between T1DM and T2DM patients with their risk of fracture at various sites.

## Supplementary Information

**Additional file 1. **Funnel plot.

## Data Availability

All data generated or analysed during this study are included in this published article and its supplementary information files.

## Abbreviations

DM: Diabetes mellitus
T1DM: Type 1 diabetes mellitus
T2DM: Type 2 diabetes mellitus
OR: Odds ratio
CI: Confidence interval
ROR: Ratio of odds ratio
NOF: National Osteoporosis Foundation
BMI: Ody mass index
NOS: Newcastle–Ottawa Scale
