# Supplementary material for: Differences in the roles of types 1 and 2 diabetes in the susceptibility to the risk of fracture: a systematic review and meta-analysis
Source: Diabetol Metab Syndr. 2021 Aug 16;13:84. doi: 10.1186/s13098-021-00687-8 (PMC8369647; doi:10.1186/s13098-021-00687-8)
Supplement: Supplementary file 1 — Additional file 1. Funnel plot. [file 13098_2021_687_MOESM1_ESM.docx]

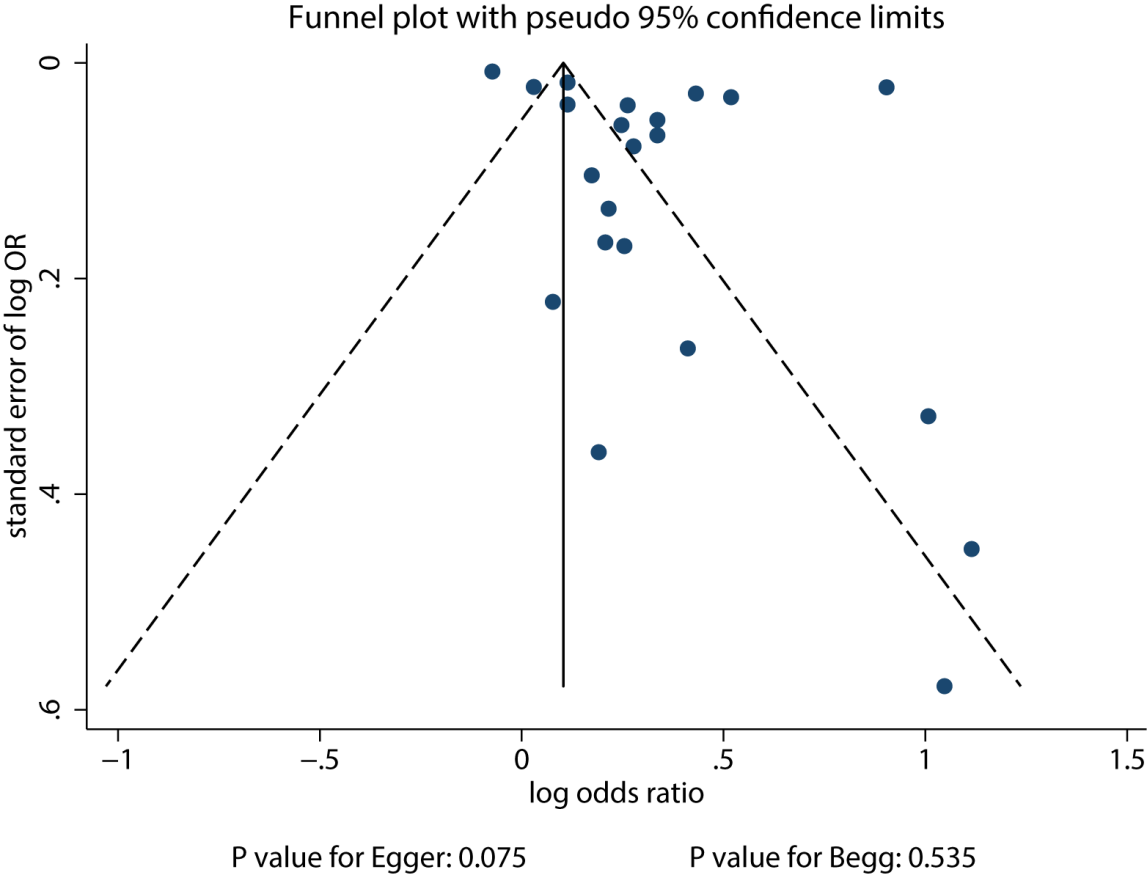


Figure S1. Funnel plot for all fracture


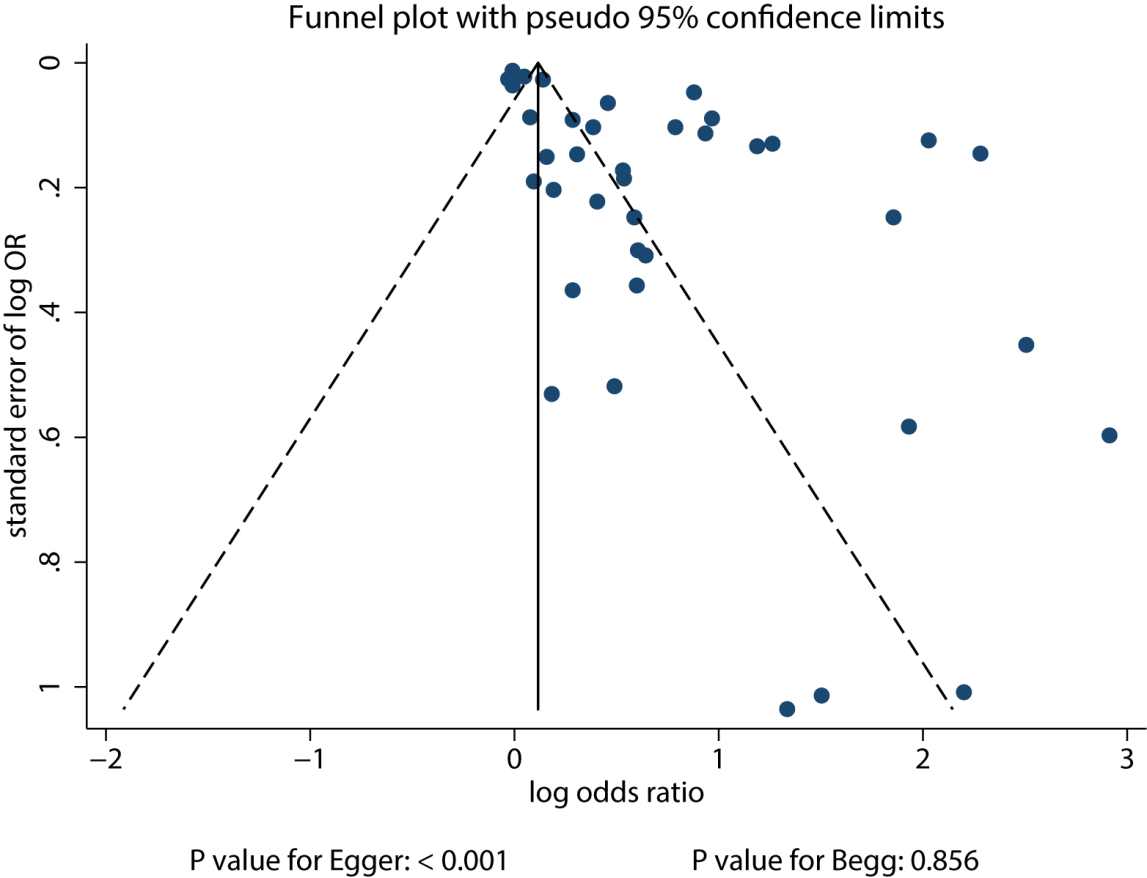


Figure S2. Funnel plot for hip fracture


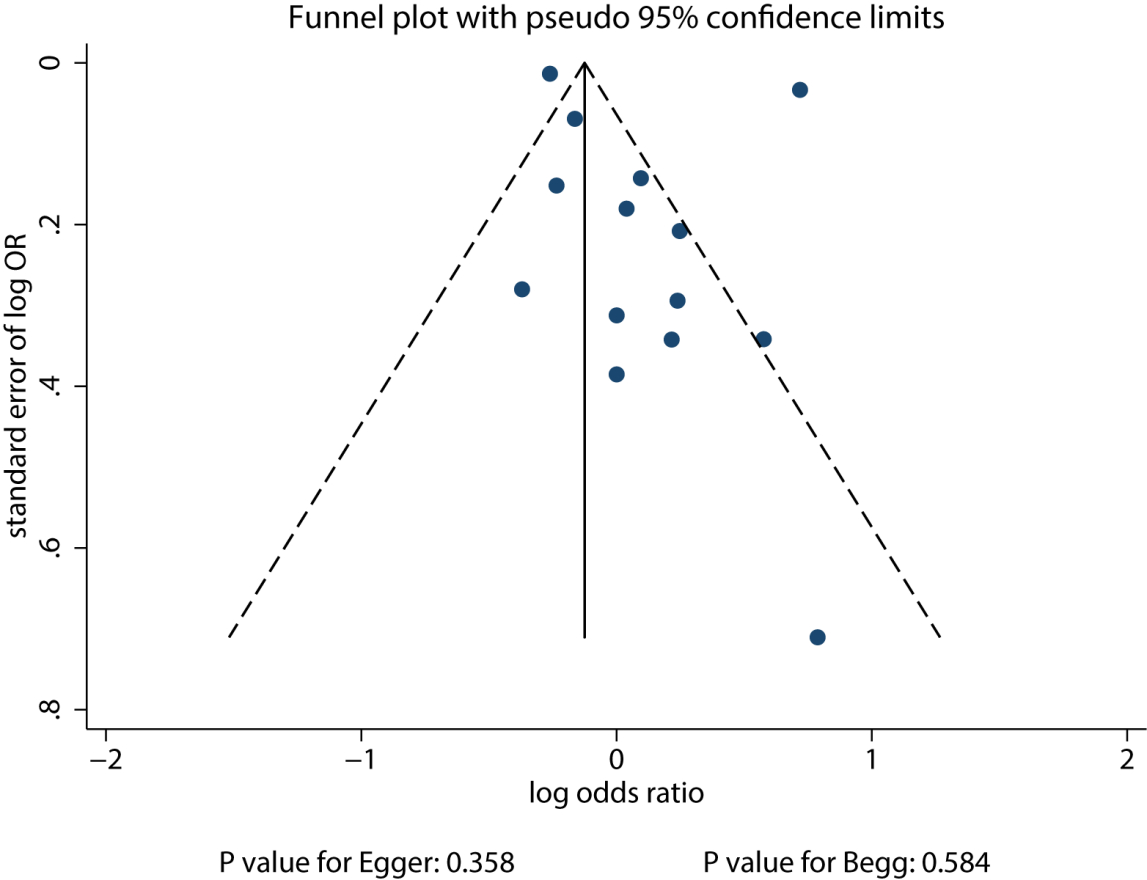


Figure S3. Funnel plot for distal forearm fracture


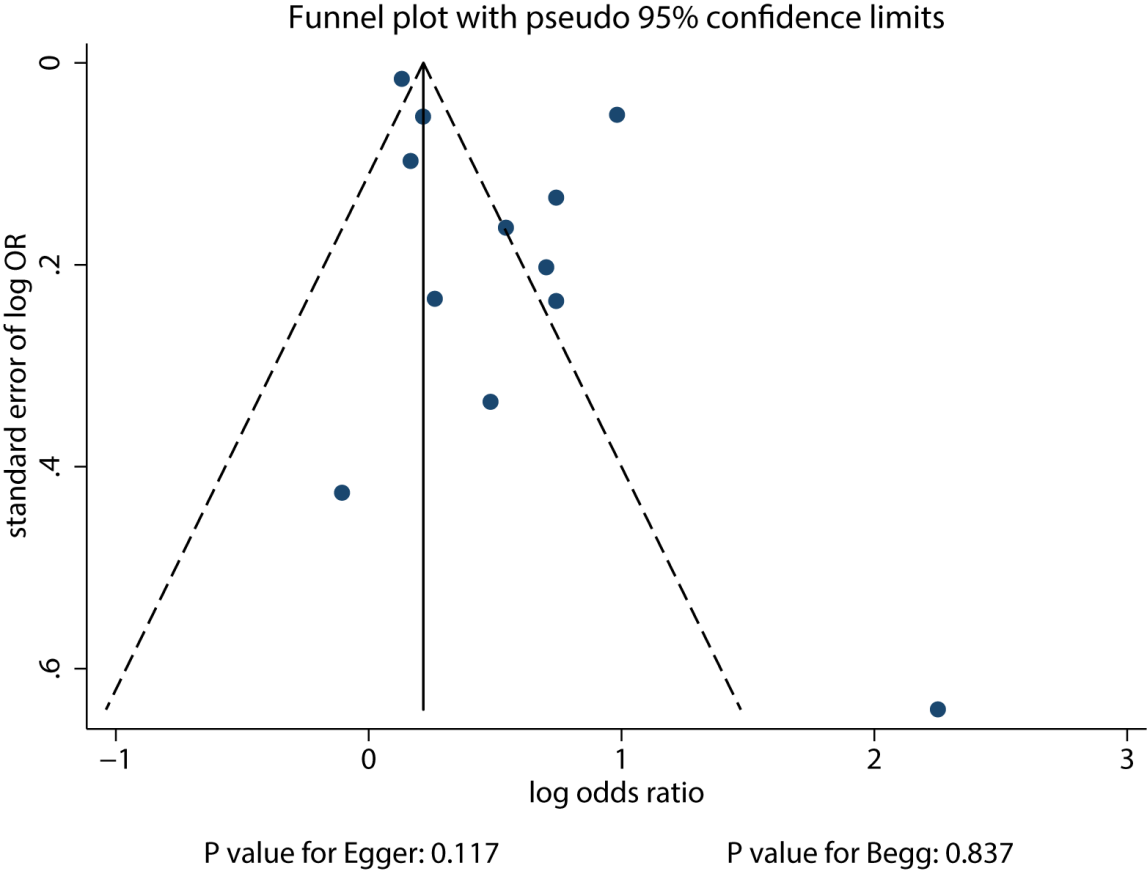


Figure S4. Funnel plot for upper arm fracture


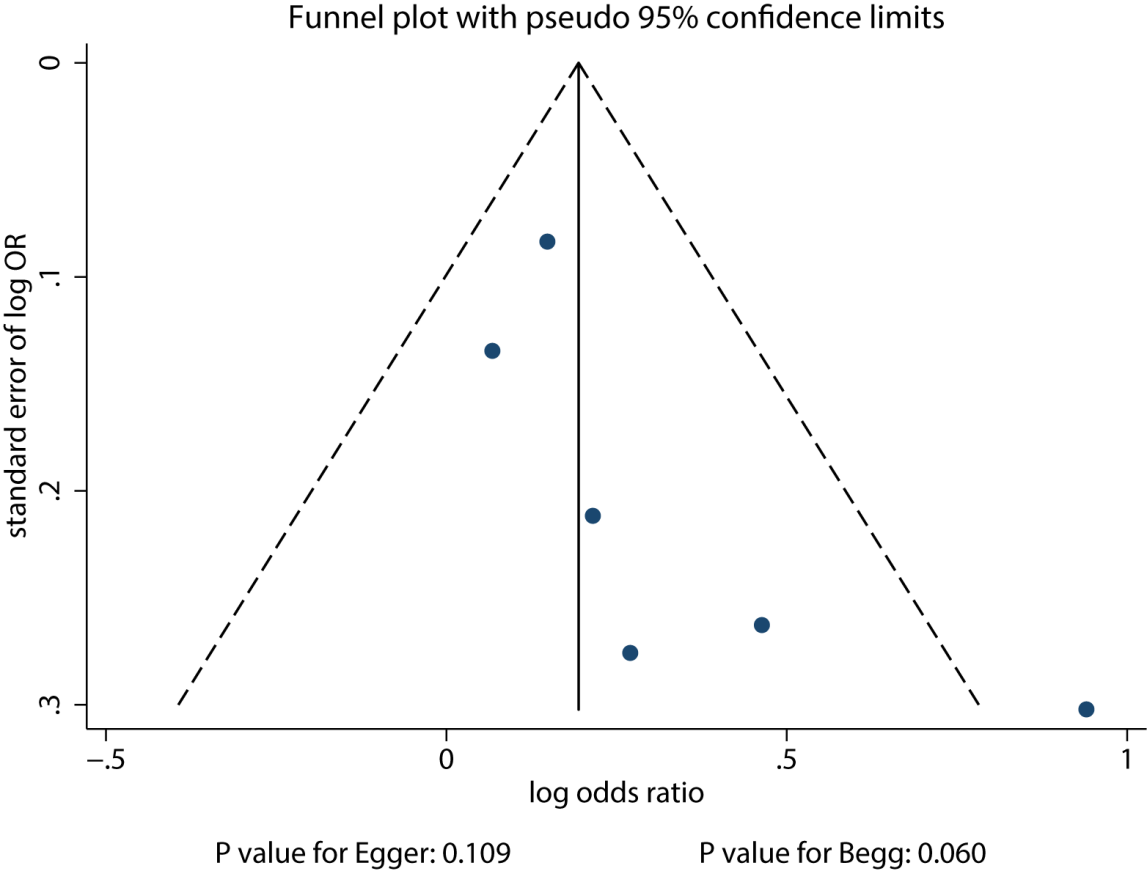


Figure S5. Funnel plot for ankle fracture


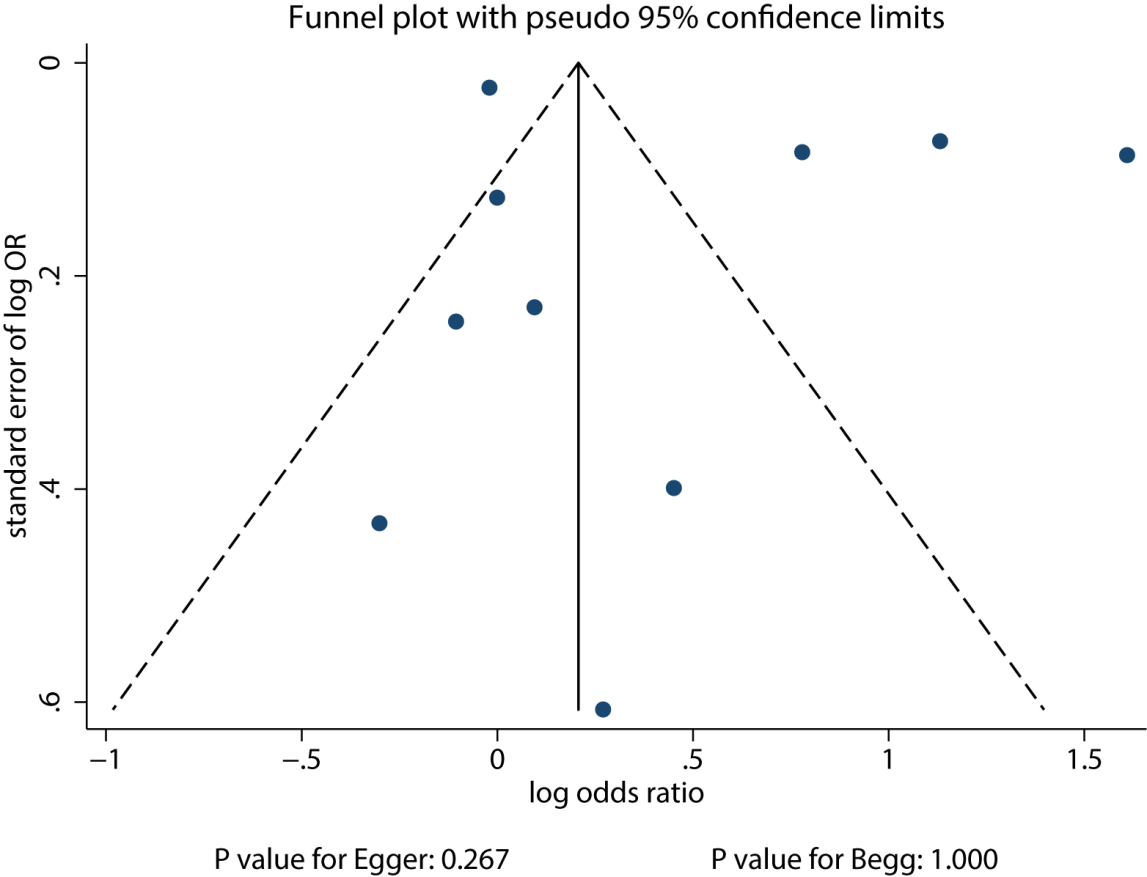


Figure S6. Funnel plot for vertebrae fracture
